# Knowledge, Perceptions, and Practices Regarding Pediatric Tuberculosis: Cross-Sectional Study Among Guardians and Health Care Providers in Kabondo Dianda Health Zone, Democratic Republic of the Congo

**DOI:** 10.2196/86657

**Published:** 2026-05-19

**Authors:** Jeanine Diur Yav, Armand Mutwadi, Albert Kalonji, Fiston Ilunga Mbayo, Pacifique Kanku wa Ilunga, Pascal Geri Madragule, John Ditekemena Dinanga

**Affiliations:** 1 Public Health Kabondo Dianda General Reference Hospital Kabondo Dianda, Haut Lomami The Democratic Republic of The Congo; 2 School of Public Health Medicine University of Kinshasa Kinshasa, Kinshasa The Democratic Republic of The Congo; 3 Public Health Malemba Nkulu General Reference Hospital Malemba Nkulu, Haut-Lomami The Democratic Republic of The Congo; 4 School of Public Health University of Malemba Nkulu Malemba Nkulu, Haut-Lomami The Democratic Republic of The Congo; 5 School of Public Health Faculty of Medicine University of Kamina Kamina, Haut-Lomami The Democratic Republic of The Congo

**Keywords:** tuberculosis, pediatric tuberculosis, knowledge-attitudes-practices, guardians, health care providers, Kabondo Dianda health zone, Democratic Republic of the Congo, cross-sectional study, public health, diagnostic practices, preventive knowledge, community perceptions

## Abstract

**Background:**

Tuberculosis (TB) remains one of the leading infectious causes of morbidity and mortality worldwide, with children representing a particularly vulnerable group. In the Democratic Republic of the Congo, pediatric TB continues to be underdiagnosed and underreported, particularly in rural health zones such as Kabondo Dianda.

**Objective:**

This study aimed to assess the knowledge, perceptions, and practices of guardians and health care providers regarding pediatric TB in Kabondo Dianda health zone by using the knowledge, attitude, and practice framework.

**Methods:**

A descriptive cross-sectional study was conducted in 5 diagnostic and treatment centers between January 2020 and December 2022. Guardians of children diagnosed with TB (163/163, 100%) were recruited exhaustively through facility registers and community health workers, while health care providers (27/27, 100%) were included through convenience sampling. Data were collected using a structured questionnaire adapted from national and World Health Organization guidelines. The instrument was pretested with 15 guardians and 5 health care providers in a neighboring health zone to ensure clarity, cultural appropriateness, and reliability; internal consistency was confirmed with Cronbach α (α=0.82). Composite scores were calculated for knowledge, perceptions, and practices. Descriptive statistics and chi-square and Fisher exact tests were applied, with significance set at *P*<.05.

**Results:**

Guardians demonstrated partial biomedical knowledge: cough was widely recognized as a symptom (123/163, 75.5%); however, misconceptions persisted, with 29% (47/163) attributing TB to supernatural causes and 52% (85/163) believing domestic animals were vectors. Preventive knowledge was limited, with only 26.4% (43/163) mentioning the Bacille Calmette-Guérin vaccination. Care seeking was often delayed, with 38.7% (63/163) consulting only after treatment failure and 25.8% (42/163) after symptom worsening. Health care providers showed higher knowledge scores but limited diagnostic engagement: only 18.5% (5/27) reported routine use of GeneXpert, and 3.7% (6/163) of pediatric cases received HIV screening. Misconceptions were significantly associated with lower knowledge scores among guardians (*P*=.03).

**Conclusions:**

Pediatric TB underreporting in Kabondo Dianda reflects both systemic and behavioral determinants. Applying the knowledge, attitude, and practice framework highlights how cultural beliefs, limited preventive knowledge, and weak diagnostic engagement interact to hinder early detection and management. Strengthening community education, training health care providers on latent TB and HIV coinfection, and improving diagnostic infrastructure are essential to reduce morbidity and mortality among children in rural Democratic Republic of the Congo.

## Introduction

### Background

Tuberculosis (TB) remains one of the leading infectious causes of morbidity and mortality worldwide. According to the World Health Organization (WHO) Global Tuberculosis Report 2024, an estimated 10.6 million people developed TB in 2023, including 1.2 million children aged <15 years [1]. Children represent a particularly vulnerable group; however, pediatric TB continues to be underdiagnosed and underreported due to its paucibacillary nature and the limited sensitivity of available diagnostic tools [2].

In sub-Saharan Africa, weak health systems, limited health care provider capacity, and low community awareness exacerbate the burden of pediatric TB [3]. In the Democratic Republic of the Congo, TB incidence remains high, with over 220 cases per 100,000 inhabitants in 2023, placing the country among the 30 high-burden nations identified by WHO [1]. Despite national control efforts, pediatric TB has not received adequate attention. In Haut-Lomami province, particularly the Kabondo Dianda health zone, notification rates for pediatric TB remain persistently low (9%-15% between 2016 and 2022) [4]. This underreporting reflects both systemic barriers (limited diagnostic infrastructure and weak referral systems) and behavioral barriers (delayed care seeking and misconceptions about transmission).

Community-level misconceptions are widespread: nearly half of caregivers believe TB is not transmissible, while more than one-quarter attribute the disease to supernatural causes [4]. Knowledge of latent TB is virtually absent among both caregivers and health care providers. Although some health care providers demonstrate high knowledge scores, most remain unaware of latent TB forms, limiting their ability to implement preventive strategies [4]. Care-seeking behavior is also delayed, with many caregivers seeking care only after treatment failure or disease progression. At the facility level, diagnostic capacity is limited: HIV screening is rarely performed, and GeneXpert testing is underused [4].

To guide this study, we adopted the knowledge, attitude, and practice (KAP) framework, which posits that gaps in knowledge and distorted perceptions influence health-seeking practices and ultimately affect disease outcomes [5]. Applying this framework allows us to analyze how caregiver beliefs and health care provider practices interact to shape pediatric TB detection and management in rural Democratic Republic of the Congo.

### Knowledge Gap

While pediatric TB is recognized as a neglected issue globally, few studies have systematically assessed the interplay of caregiver perceptions, health care provider practices, and systemic barriers in rural Congolese settings. Existing literature highlights underreporting but does not adequately explain the behavioral and cultural determinants underlying delayed diagnosis and poor management [6,7].

### Objectives

This study aims to assess the knowledge, perceptions, and practices of guardians of children and health care providers regarding pediatric TB in Kabondo Dianda health zone using the KAP framework. Specifically, we seek to identify misconceptions and cultural beliefs influencing care seeking, evaluate health care provider diagnostic and preventive practices, and highlight systemic gaps that hinder early detection. By addressing these dimensions, the study contributes to evidence-based strategies for strengthening pediatric TB control and reducing morbidity and mortality among children in resource-limited environments.

## Methods

### Study Design and Setting

This study used a descriptive cross‑sectional design, which is appropriate for generating baseline evidence on knowledge, perceptions, and practices without testing causal hypotheses. The research was conducted in the Kabondo Dianda health zone, Haut‑Lomami province, Democratic Republic of the Congo. The zone comprises 19 health areas, of which 5 were designated as diagnostic and treatment centers (CSDTs) by the Provincial TB Coordination Unit. These sites were selected for their strategic location, patient volume, and relevance to pediatric TB surveillance. Data collection took place between November 25 and 30, 2022, covering pediatric TB cases diagnosed from January 2020 to December 2022. As the study period extended over 3 years, recall bias was anticipated and is explicitly acknowledged as a limitation of descriptive studies.

### Recruitment and Study Population

Participants were recruited from the 5 CSDTs officially designated by the Provincial TB Coordination Unit in the Kabondo Dianda health zone. Recruitment of guardians was exhaustive: all caregivers of children aged <15 years who had been diagnosed with pulmonary TB between January 2020 and December 2022 were identified through facility registers and community health workers. Of the 166 eligible guardians, 163 were successfully enrolled, while 3 were excluded due to permanent displacement following local flooding. This approach ensured near‑complete coverage of the target population and minimized selection bias.

Health care providers were recruited using convenience sampling. All personnel directly involved in TB diagnosis and management at the selected facilities were invited to participate, including medical directors, nurses, laboratory technicians, and TB focal points. A total of 27 health care providers were enrolled, representing the full spectrum of staff engaged in pediatric TB services. Although exploratory in nature, this sample reflects the operational realities of TB care in the health zone.

Eligibility criteria for guardians required being the primary caregiver of a child aged <15 years diagnosed with pulmonary TB in one of the selected CSDTs during the study period. Health care providers were eligible if they were actively assigned to the selected facilities at the time of data collection and directly responsible for TB‑related services. Exclusion criteria included permanent relocation outside the health zone or refusal to provide informed consent. By combining exhaustive recruitment of guardians with comprehensive inclusion of available health care providers, the study population was designed to capture both community‑level experiences and facility‑based practices relevant to pediatric TB detection and management.

### Data Collection

Data were collected using a structured questionnaire administered via KoboCollect, adapted from national TB guidelines and WHO recommendations. The tool was pretested on 15 guardians and 5 health care providers in a neighboring health zone to ensure clarity, cultural appropriateness, and reliability. Internal consistency was assessed using Cronbach α, which yielded a coefficient of 0.82. The questionnaire contained closed‑ended questions covering sociodemographic characteristics, knowledge of TB transmission and prevention, perceptions of severity and stigma, and care‑seeking practices. Health care provider–specific items assessed diagnostic capacity, GeneXpert use, HIV screening, and referral procedures.

### Instrument Validation

Prior to data collection, the questionnaire was subjected to a pretest to ensure clarity, cultural appropriateness, and reliability. The tool was administered to a sample of 15 guardians and 5 health care providers in a neighboring health zone. Feedback from this pilot exercise was used to refine wording and improve comprehension. Internal consistency was assessed using Cronbach α, which yielded a coefficient of 0.82, indicating good reliability. This validation process confirmed that the instrument was suitable for use in the study population and aligned with WHO recommendations for KAP surveys [1].

### Variables and Operational Definitions

Independent variables included type of facility, health care provider profile (qualification and years of experience), guardian characteristics (age, education, occupation, and household size), availability of diagnostic tools, and accessibility of services. Dependent variables included knowledge level, perceptions, practices such as timely versus delayed care seeking, and health care provider performance in screening, referral, and counseling. High knowledge was defined as at least 75% correct responses, based on WHO KAP survey guidelines. Delayed care seeking was defined as seeking care only after symptom worsening or treatment failure. Diagnostic engagement referred to the use of GeneXpert or HIV testing for pediatric TB cases.

### Handling of Missing Data

Given the descriptive nature of the study, missing data were managed using a complete‑case analysis approach. Partially missing responses were excluded from composite score calculations. Sensitivity analyses confirmed that exclusion did not alter key descriptive findings.

### Data Analysis

Data were analyzed using SPSS (version 25.0; IBM Corp). Descriptive statistics such as frequencies, proportions, medians, and IQRs were calculated to summarize participant characteristics and responses. Exploratory associations were examined using χ²and Fisher exact tests, with statistical significance set at *P*<.05. Composite scores were developed to assess knowledge and perception levels, consistent with descriptive study standards.

### Ethical Considerations

Ethics approval for this study was obtained from the ethics committee of the School of Public Health, University of Kinshasa (approval number ESP/CE/205/2024). Written authorization was also granted by the head of the health division of Haut‑Lomami Province and the chief medical officer of the Kabondo Dianda health zone. Investigators consistently carried these authorizations during fieldwork and presented them whenever requested by local authorities or participants. Written informed consent was obtained from all participants prior to their inclusion in the study.

## Results

### Overview

The sociodemographic characteristics of guardians and health care providers are shown in Table 1. Of the 163 guardians, 82 (50.3%) were female, with a median age was 37 (IQR 30-43) years. Farming was the predominant occupation (n=88, 54%), and nearly half lived in households with 5 to 9 members (n=80, 49.1%). Of the 27 health care providers, 22 (81.5%) were male, with a median age of 35 (IQR 10) years. All were officially employed, but only 8 (29.6%) had completed secondary school and 1 (3.7%) had attained university-level education.

**Table 1:** Sociodemographic characteristics of the guardians and health care providers.

Characteristic	Guardians (n=163)	Health care providers (n=27)
Female sex, n (%)	82 (50.3)	5 (18.5)
Age (years), median (IQR)	37 (13)	35 (10)
Farming occupation, n (%)	88 (54)	0 (0)
Household size: 5-9, n (%)	80 (49.1)	12 (44.4)

### Knowledge of TB Transmission

Knowledge of TB transmission is shown Table 2. Coughing was correctly identified as a transmission route by 75.5% (123/163) guardians and 88.9% (24/27) health care providers. Health care providers demonstrated broader awareness, with 20 (74.1%) citing sneezing, 18 (66.7%) mentioning speaking, and 16 (59.3%) recognizing laughing. Guardians showed limited awareness beyond coughing, with only 36 (22.1%) mentioning sneezing and 12 (7.4%) mentioning laughing. Differences between guardians and health care providers were statistically significant for sneezing (*P*=.001) and laughing (*P*<.001).

**Table 2:** Knowledge of tuberculosis transmission.

Transmission mode	Guardians (n=163), n (%)	Health care providers (n=27), n (%)	*P* value
Coughing	123 (75.5)	24 (88.9)	.12
Sneezing	36 (22.1)	20 (74.1)	.001
Speaking	55 (33.7)	18 (66.7)	.01
Laughing	12 (7.4)	16 (59.3)	<.001

### Knowledge of TB Prevention

Knowledge of TB prevention is shown in Table 3. Preventive knowledge was markedly higher among health care providers. All 100% (27/27) identified good nutrition, 26 (96.3%) cited Bacille Calmette-Guérin (BCG) vaccination, and 19 (70.4%) mentioned respiratory hygiene. Guardians reported lower knowledge, with 26.4% (43/163) citing good nutrition, 43 (26.4%) mentioning BCG vaccination, and 18 (11%) citing covering the mouth or avoiding overcrowding. Differences between groups were statistically significant for BCG vaccination and respiratory hygiene (*P*<.001).

**Table 3:** Knowledge of tuberculosis prevention.

Prevention method	Guardians (n=163), n (%)	Health care providers (n=27), n (%)	*P* value
Good nutrition	43 (26.4)	27 (100)	<.001
BCG^a^ vaccination	43 (26.4)	26 (96.3)	<.001
Covering mouth	18 (11)	19 (70.4)	<.001

^a^BCG: Bacille Calmette-Guérin.

### Perceptions of TB Transmission

Among health care providers, 89% (24/27) correctly perceived TB as communicable and transmitted by coughing. In contrast, only 55% (90/163) guardians recognized TB as communicable. Misconceptions persisted: 47 (29%) guardians attributed TB to supernatural causes, and 85 (52%) believed domestic animals were vectors. Even among health care providers, 3 (11%) cited evil spirits, and 25 (93%) implicated animals.

### Care-Seeking Practices

Formal health facilities were the first point of care for 49.1% (80/163) guardians and 70.4% (19/27) health care providers. Guardians frequently sought care at pharmacies (n=43, 26.4%) or traditional healers (n=32, 19.6%). Timing of care seeking showed delays: 63 (38.7%) guardians consulted only after treatment failure, and 42 (25.8%) after symptom worsening. Among health care providers, 9 (33.3%) reported initiating care after deterioration. Reasons for choosing facilities included free effective treatment (n=60, 36.8% guardians and n=19, 70.4% health care providers) and accessibility (n=39, 23.9% guardians).

### Diagnostic Practices

Despite the availability of diagnostic tools, use was limited. Only 3.7% (6/163) pediatric cases received HIV screening, and 18.5% (5/27) health care providers reported routine use of GeneXpert. These findings confirm systemic gaps in diagnostic engagement.

## Discussion

### Principal Findings

This descriptive study revealed substantial gaps in knowledge, perceptions, and practices regarding pediatric TB among guardians and health care providers in the Kabondo Dianda health zone. Guardians demonstrated limited awareness of transmission routes beyond coughing, and misconceptions such as attributing TB to supernatural causes or domestic animals were common. Health care providers showed broader biomedical knowledge but still reported beliefs inconsistent with scientific evidence. Preventive knowledge was markedly higher among health care providers, particularly regarding nutrition and BCG vaccination, whereas guardians cited these measures less frequently. Care‑seeking practices were delayed, with many guardians consulting health services only after treatment failure or symptom worsening. Diagnostic engagement remained low, with limited use of GeneXpert and HIV screening.

### Comparison With Prior Studies

These findings align with previous research in sub‑Saharan Africa, which has documented persistent misconceptions about airborne transmission and cultural beliefs attributing TB to nonbiomedical causes [8,9]. Similar studies in rural contexts have shown that delayed care seeking is often driven by poverty, geographic isolation, and reliance on traditional healers [10]. The low use of GeneXpert and HIV screening mirrors national reports from the Democratic Republic of the Congo, where diagnostic infrastructure exists but is underused due to limited training and supervision [11]. The disparities between guardians and health care providers in knowledge of prevention echo findings from other African settings, where community‑level education remains insufficient [12].

### Implications for Practice and Policy

The results underscore the urgent need to strengthen pediatric TB control strategies in rural Democratic Republic of the Congo. Community education campaigns should specifically target misconceptions, emphasizing airborne transmission and the importance of early care seeking. Health care provider training must address latent TB, HIV coinfection, and systematic use of GeneXpert. Health system strengthening is required to improve diagnostic infrastructure, referral pathways, and integration of national guidelines into routine practice. Finally, culturally sensitive communication strategies should be developed, respecting local beliefs while promoting evidence-based practices. Collaboration between community health workers, diagnostic centers, and local authorities can enhance case tracking and reduce stigma.

### Limitations

This study has several limitations inherent to descriptive cross‑sectional designs. Recall bias may have affected guardian responses, given the 3‑year study period. Selection bias is possible due to convenience sampling of health care providers, which limits representativeness. Self‑reported data may be subject to social desirability bias. Moreover, the study was conducted in 5 diagnostic centers within Kabondo Dianda and may not reflect practices in other health zones. Despite these limitations, exhaustive sampling of guardians and inclusion of all active health care providers strengthen internal validity.

### Conclusions

This descriptive study conducted in Kabondo Dianda health zone highlights persistent gaps in knowledge, perceptions, and practices related to pediatric TB among guardians and health care providers. While health care providers demonstrated relatively higher biomedical knowledge, misconceptions remained, and diagnostic engagement was limited. Guardians showed restricted awareness of transmission and prevention, with delayed care-seeking behaviors, contributing to underreporting and late diagnosis.

By applying the KAP framework, the study underscores how cultural beliefs, limited preventive knowledge, and systemic barriers interact to hinder early detection and effective management of pediatric TB in rural Democratic Republic of the Congo. These findings emphasize the need for targeted community education, health care provider training on latent TB and HIV coinfection, and improved diagnostic infrastructure.

Although limited by recall bias, selection bias, and reliance on self-reported data, this study provides valuable baseline evidence for strengthening pediatric TB control strategies in resource-constrained settings. Future research should explore interventions that integrate culturally sensitive communication with systematic diagnostic practices to reduce morbidity and mortality among children.

## Data Availability

The anonymized dataset used for this study is available from the corresponding author upon reasonable request.

## Abbreviations

BCG: Bacille Calmette-Guérin
CSDT: diagnostic and treatment center
KAP: knowledge, attitude, and practice
TB: tuberculosis
WHO: World Health Organization
